# Activation of chloride transport in CF airway epithelial cell lines and primary CF nasal epithelial cells by S-nitrosoglutathione

**DOI:** 10.1186/1465-9921-7-124

**Published:** 2006-10-05

**Authors:** Zhanna Servetnyk, Jelena Krjukova, Benjamin Gaston, Khalequz Zaman, Lena Hjelte, Godfried M Roomans, Anca Dragomir

**Affiliations:** 1 Department of Medical Cell Biology, Uppsala University, Uppsala, Sweden; 2 Department of Pediatrics, University of Virginia Health System, Charlottesville, VA, USA; 3 Stockholm CF Center, Karolinska University Hospital Huddinge, Stockholm, Sweden

## Abstract

**Background:**

It has been suggested that low μM concentrations of S-nitrosoglutathione (GSNO), an endogenous bronchodilator, may promote maturation of the defective cystic fibrosis (CF) transmembrane conductance regulator (CFTR). Because nitric oxide (NO) and GSNO levels appear to be low in the CF airway, there is an interest in the possibility that GSNO replacement could be of therapeutic benefit in CF.

**Methods:**

The effect of GSNO on chloride (Cl^-^) transport was investigated in primary nasal epithelial cells obtained from CF patients homozygous for the delF508 mutation, as well as in two CF cell lines (CFBE and CFSME), using both a fluorescent Cl^- ^indicator and X-ray microanalysis. Maturation of delF508 CFTR was determined by immunoblotting.

**Results:**

Treatment with 60 μM GSNO for 4 hours increased cAMP-induced chloride efflux in nasal epithelial cells from 18 out of 21 CF patients, but did not significantly affect Cl^- ^efflux in cells from healthy controls. This Cl^- ^efflux was confirmed by measurements with a fluorescent Cl^- ^indicator in the CFBE and CFSME cell lines. The effect of GSNO on Cl^- ^efflux in CFBE cells could be inhibited both by a specific thiazolidinone CFTR inhibitor (CFTR_inh_-172) and by 4,4'-diisothiocyanatodihydrostilbene-2,2'-disulfonic acid (H_2_DIDS). X-ray microanalysis showed that, following 4 hours incubation with 60 μM GSNO, cAMP agonists caused a decrease in the cellular Cl^- ^concentration in CFBE cells, corresponding to Cl^- ^efflux. GSNO exposure resulted in an increase in the protein expression and maturation, as shown by immunoblot analysis. GSNO did not increase the cytosolic Ca^2+ ^concentration in cultured airway epithelial cells.

**Conclusion:**

Previous studies have suggested that treatment with GSNO promotes maturation of delF508-CFTR, consistent with our results in this study. Here we show that GSNO increases chloride efflux, both in the two CF cell lines and in primary nasal epithelial cells from delF508-CF patients. This effect is at least in part mediated by CFTR. GSNO may be a candidate for pharmacological treatment of the defective chloride transport in CF epithelial cells.

## Background

Cystic Fibrosis (CF) is a common life-shortening inherited disorder [1]. The disease is caused by mutations in the gene for the cystic fibrosis transmembrane conductance regulator (CFTR), a cAMP-regulated chloride channel present in the apical membrane of epithelia in many organs, including airways [2].

CFTR is synthesized in the endoplasmic reticulum (ER) and transported to the Golgi complex where, after N-glycosylation, it becomes a mature protein that via the secretory pathway reaches the plasma membrane. The folding of CFTR occurs by complex interactions between newly synthesized CFTR and chaperones such as heat shock cognate (Hsc) 70 and, later, the ER chaperones calnexin and heat shock protein (Hsp70) [3-5]. The processing of CFTR in the ER is an inefficient process followed by rapid degradation of about 75% of nascent wild type CFTR. The high rate of degradation is believed to be an intrinsic property of CFTR [6]. Recently, Yoo et al. [7] reported an alternative pathway of CFTR trafficking between ER and Golgi complex that may also affect CFTR expression in the cell membrane.

The most common CFTR mutation in the CF population, delF508, leads to the expression of a protein with a missing phenylalanine residue at the 508 position in the nucleotide binding domain 1 (NBD 1) [2]. Deletion of F508 interferes with proper folding and trafficking of CFTR-delF508 from ER to the cell surface [8]. However, if CFTR-delF508 reaches the cell membrane, it is able to mediate partial Cl^- ^conductance even though the channel exhibits altered kinetics [9]. This suggests a possible therapeutic strategy for CF treatment based on increasing the efficiency of folding and intracellular processing of this mutant [9,10].

The endogenous bronchodilator, S-nitrosoglutathione (GSNO) has been proposed as a possible pharmacological remedy that reverses the CFTR-ΔF508 maturation defect as detected by immunoblot [11], causes a 4-fold increase in CFTR-mediated chloride efflux and promotes apical location of CFTR in cultured CF airway epithelial cells [12]. Preliminary evidence suggests that GSNO can affect CFTR expression and maturation through several mechanisms, including 1) a transcriptional effect involving specificity protein (Sp)3 [11]; and 2) post-translational effects mediated by an effect on cysteine string protein expression, on heat shock cognate 70 S-nitrosylation [13] and other effects. However, the effect of GSNO on native cells taken from CF patients or primary cell cultures has not yet been investigated; and pharmacological evidence that the Cl^- ^efflux measured involves CFTR has been incomplete. Further, high μM levels of GSNO could have adverse effects on CFTR of inhibiting its transcription, augmenting its degradation and inhibiting its function. Therefore, it is important to determine the precise effect of GSNO on airway cells taken directly from CF patients. Here, we show that GSNO increases maturation and function of delF508 CFTR using both airway epithelial cell lines and primary nasal epithelial cells from CF patients.

## Methods

### Individuals and genotypes

Nasal epithelial cells were obtained by brush biopsy from patients with CF homozygous for the delF508 mutation (n = 21) and healthy volunteers with no clinical signs of CF (n = 8). Genotypes of the patients participating in the study were determined by established DNA molecular techniques[14]. The study was approved by the Ethical Committee of Karolinska University Hospital and the participants had given informed consent.

### Obtaining nasal cells by brush biopsy

Nasal brushing was performed as described elsewhere [15]. Briefly, an interdental brush with 2.5 or 3.0 mm bristles (Apoteket AB, Stockholm, Sweden) was used to scrape along the inferior nasal turbinate and lateral nasal wall. Brushes with cells were immediately placed into 1.5 ml of Eagle's minimal essential medium (EMEM) with Glutamax (SVA, Uppsala, Sweden) supplemented with 10% fetal bovine serum, 100 U/ml penicillin, and 100 μg/ml streptomycin sulfate at room temperature. When cells needed to be transported to the laboratory the tube was kept at 4°C for no longer than 3 hours. Cells were removed from the brush by passing it up and down through a disposable P200 pipette tip (Treff, Degersheim, Switzerland) with the end of the tip cut off. Cells were kept at 37°C in a 5% CO_2_-humidified incubator overnight.

### Chemicals and solutions

The fluorescent probe N-(ethoxycarbonylmethyl)-6-methoxyquinolinium bromide (MQAE) nigericin and 4,4'-diisothiocyanatodihydrostilbene-2,2'-disulfonic acid disodium salt hydrate (H_2_DIDS) were obtained from Molecular Probes (Eugene, OR, U.S.A); tributyltin acetate from Aldrich-Chemie (Steinheim, Germany); the NO-donor GEA3162 (5-amino-3-(3,4-dichlorophenyl)1,2,3,4-oxatriazolium) (Moilanen et al., 1993) was from Alexis Biochemicals (San Diego, CA, USA); forskolin, 3-isobutyl-1-methylxantine (IBMX), and ATP were from Sigma (St Louis, MO, U.S.A). Thiazolidinone CFTR inhibitor (CFTR_inh_-172) was a generous gift of Prof. A. Verkman, San Francisco, CA, U.S.A.

Standard Ringer's solution (SR) consisted of: 140 mM NaCl, 5 mM KCl, 5 mM (2-hydroxyethyl)-*N*-piperazine *N*-2 ethanesulphonic acid (HEPES), 1 mM MgCl_2 _and 5 mM glucose, pH 7.4. In chloride-free Standard Ringer's solution the chloride salts were exchanged for nitrate salts. K^+^-rich calibration buffers were made by mixing of 150 mM chloride solution (120 mM KCl, 1 mM MgCl_2_, 27 mM NaCl, 5 mM glucose and 5 mM HEPES), and 150 mM NO_3 _^- ^solutions (chloride exchanged for nitrate) to obtain the desired concentrations of chloride.

### Cell culture

The CFBE41o- CF bronchial epithelial (homozygous for the delF508 mutation) and the CFSMEo- CF submucosal gland epithelial (delF508/2QX) cell lines, generous gifts from Dr. D. Gruenert, San Francisco, CA, U.S.A., were cultured in EMEM with Glutamax (SVA) supplemented with 10% fetal bovine serum (Gibco, Paisley, U.K.), 100 U/ml penicillin, and 100 μg/ml streptomycin sulfate at 37°C in a 5% CO_2_-humidified incubator. The medium was changed twice weekly.

### Intracellular chloride measurements

Nasal epithelial cells were made to attach to glass cover slips by treating slides with BD Cell-Tak (BD Bioscience, Bedford, MA, U.S.A). CFBE and CFSME cells were grown to confluence on glass cover slips. Prior to Cl^- ^efflux measurements, the cell lines and the nasal epithelial cells were treated with 60 μM GSNO for a total of 4 hours at 37°C. The treatment was performed by adding freshly made GSNO solution every 30 minutes, by exchanging the incubation solution. The cells were loaded with 10 mM MQAE for 2 h, rinsed in SR solution and the cover slips were placed as bottom in a perfusion chamber on the stage of an inverted microscope (Nikon, Diaphot, Japan). The temperature was kept at 37°C by heating the chamber holder and the objective separately. A 75 W xenon lamp and a monochromator, part of a Quanticell 2000 image-processing system (VisiTech International, Sunderland, U.K.), provided excitation light at 355 nm (12 nm bandwidth). The emission was measured at 460 nm (30 nm bandwidth) using a CCD camera. Cells were bathed in SR. Images were captured for 16 ms every 3–8 seconds as described elsewhere [16].

The basal Cl^- ^efflux was measured during the exposure of cells loaded with MQAE to a chloride gradient. Chloride efflux was induced by changing from 150 mM chloride buffer to a chloride-free buffer with NO_3 _^- ^as the substituting anion without stimulating agents. The basal efflux was compared to that obtained in the presence of cAMP agonists (20 μM forskolin and 50 μM IBMX) under similar conditions. The agonists or blockers were added 1 min before the change of buffer.

For the nasal epithelial cells, the fluorescence was expressed as relative change (F_rel_) in fluorescence (F) to the initial fluorescence at the beginning of the experiment (F_0_) according to

F_rel _= F/F_0 _× 100 (%)     (1)

The relative chloride efflux rate (J_Cl-_) was determined from the first derivative of F_rel _with time (ΔF_rel_/Δt), percent/s. The efflux via CFTR was determined by subtracting the basal relative efflux (J_basal_) from the cAMP-stimulated relative rate (J_cAMP_), according to:

J_CFTR _= J_cAMP _- J _basal _    (2)

The chloride measurements on nasal epithelial cells obtained from healthy individuals were performed on the day when the biopsy had been taken (day 0). The nasal cells from CF patients were kept overnight in the incubator in order to perform the 4 h GSNO incubation along with chloride measurements during the same day. Therefore, chloride studies were conducted on the next day (day 1). To verify compatibility of the results, the chloride efflux was measured on day 0 and day 1 on the cells from healthy volunteers and values obtained were not significantly different (data not shown). The chloride efflux from CFBE and CFSME cells was determined quantitatively as described previously [12].

For each experiment, all the cells in the optical field were analyzed (15–30 cells) and the average response was counted as one experimental data point. GraphPad Prism 4.0 software (GraphPad Software, San Diego, CA, U.S.A) was used to determine the value of the Cl^- ^efflux rate.

### Intracellular calcium measurements

Cells were grown on glass slides until confluence and loaded with the calcium-sensitive dye fura2-AM (Molecular Probes), 1–5 μM in standard Ringer's solution, for 45 min at 37°C. The cells were mounted as described above and excited by alternating 340 and 380 nm light using a filter changer under the control of In CytIm-2 software (Intracellular Imaging Corp., Cincinnati, OH, U.S.A) and a 430 nm dichroic mirror. The emission was measured through a 510 nm barrier filter with an integrating CCD camera (Cohu, Inc., San Diego, CA, U.S.A). A new rationed image (340 nm/380 nm) was obtained every second and the intracellular calcium concentration was calculated [17].

### X-ray microanalysis

CFBE and CFSME cells were grown to confluence on titanium grids covered with a carbon coated Formvar film (Merck, Darmstadt, Germany). The cells were rinsed in SR and the grid put in a 20 μl drop of SR (control) or chloride-free SR with 20 μM forskolin + 50 μM IBMX for 3 min. The cells were rinsed with cold distilled water for some seconds before they were frozen in liquid propane cooled by liquid nitrogen (-180°C) and freeze-dried overnight in vacuum at -150°C. The freeze-dried cells were covered with a conductive carbon layer before analysis. Analysis was performed in a Hitachi (Tokyo, Japan) H7100 electron microscope in the scanning-transmission electron microscopy (STEM) mode at 100 kV with an Oxford Instruments (Oxford, U.K.) ISIS energy-dispersive spectrometer system. Quantitative analysis was performed by comparing the ratio of the characteristic peak and the background under the peak (P/B) with the P/B ratios from standards consisting of a gelatin/glycerol matrix with mineral salts in known concentrations [18]. Spectra were acquired for 50 seconds and each cell was analyzed only once. The data were standardized, using the peak for phosphorus, to correct for variations in the extraneous background. A minimum of 30 cells/experiment was measured.

### Immunoblot analysis

CFBE cells were seeded at 0.5–1 × 10^6 ^cells/cm^2 ^and grown on 0.4 μm pore filters (Transwell, Corning, NY) at air-liquid interface. Immunoblot was performed as previously described (Zaman et al, 2001, 2004, 2006). Briefly, whole cell extracts were prepared in 1% Nonidet P40 (Sigma) lysis buffer containing 50 mM Tris-HCl (pH 8.0), 150 mM NaCl, 2 mM leupeptin, 1 mM aprotinin and 1 mM pepstain, 1 mM PMSF and 2 mM Na_2_VO_4 _(Boehringer Mannheim, Indianapolis, IN) were sheared through a 25-gauge needle, fractioned on a 6% SDS polyacrylamide gel in 1x electrode buffer (25 mM Tris, 192 mM glycine, 0.1% SDS at pH 8.3), transferred to nitrocellulose membranes (Bio-Rad, Hercules, CA) in Tobin Transfer Buffer (25 mM Tris, 192 mM glycine, 20% methanol, pH 8.3), blocked in Tris buffered saline-Tween 20 (TBS-T = 10 mM Tris-HCl, 150 mM NaCl, 0.05% Tween 20, 5% nonfat dried milk at pH 8.0) and probed (1 hr, RT) with 1:1000 with anti-CFTR monoclonal Ab 596 (kindly provided by Dr. J. R. Riordan, Scottsdale, AZ, USA) Blots were washed, incubated (30 min) with 1:2000 HRP-conjugated anti-mouse antibody (Pierce, Rockford, IL) in TBS-T (30 min) and visualized using Hyperfilm (Amersham Pharmacia Biotech). Per line 50 mg of protein was loaded.

### Statistical analysis

Data are shown as mean ± standard error of mean (SEM). Where appropriate a paired Student's *t *test or analysis of variance (ANOVA followed by Bonferroni test) was used to estimate the difference between treated and untreated groups. A probability value p < 0.05 was considered significant.

## Results

### GSNO increases delF508 CFTR function in primary nasal epithelial cells from CF patients

**Figure 1:**
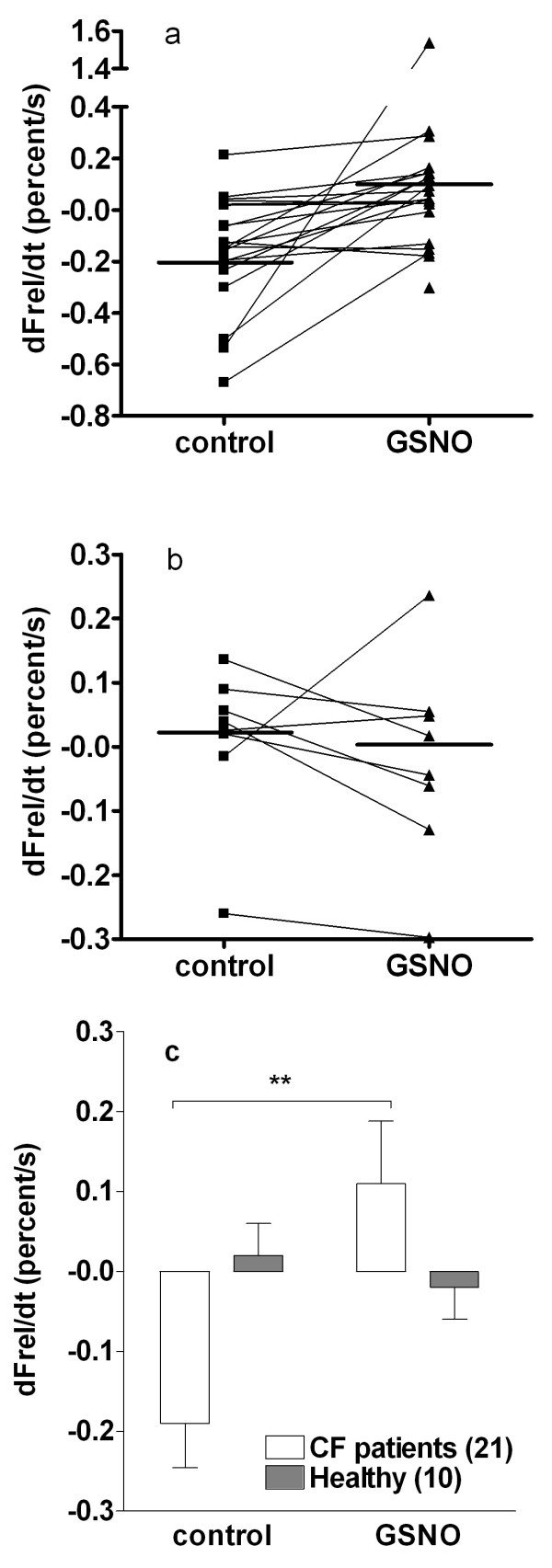
**The effect of GSNO treatment (60 μM for 4 hours) on the Cl- relative efflux rate measured in nasal epithelial cells with the MQAE fluorescence method**. Individual data from (**a**) CF patients and (**b**) healthy subjects; horizontal line indicates the mean. (**c**) Data are expressed as mean ± SEM and the number of subjects in each group is shown in the parentheses beside the legend. Significant difference from the control (paired Student's t-test) is indicated by asterisks (** p < 0.01).

In the nasal tall columnar ciliated (TCC) epithelial cells from CF patients, GSNO treatment increased the relative cAMP-dependent efflux rate (J_CFTR_) from -0.19 ± 0.06 to 0.11 ± 0.08 percent/s (see *Methods*), reaching the efflux level observed in healthy nasal columnar cells. Out of 21 patients, 18 demonstrated accelerated chloride efflux after GSNO treatment and in 3 patients this parameter did not improve (Fig. 1a, 1c). The effect of GSNO was statistically significant (p < 0.01). Chloride efflux in nasal cells from healthy persons was not affected by GSNO treatment (Fig. 1b, 1c).

### GSNO increases ΔF508 CFTR expression and maturation in ΔF508 CFTR homozygous airway epithelial cell culture

**Figure 2:**
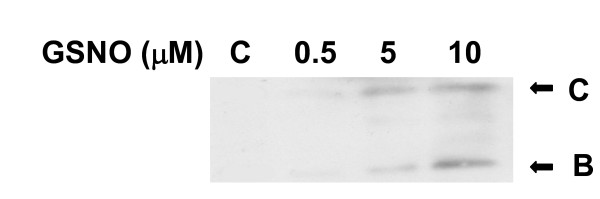
**Immunoblot analysis on homozygous ΔF508 CFBE cultured cells treated with various concentrations of GSNO**. Western blot analysis of CFTR was performed on whole cell extracts from CFBE cells using the monoclonal antibody 596, in the absence or presence of GSNO at different concentrations for 4 hours. Lane 1-control; lane 2- 0.5 μM; lane 3- 5 μM; and lane 4- 10 μM of GSNO. 50 μg of protein was loaded to each line.

Treatment with low μM concentrations of GSNO resulted in a dose-dependent increase in the expression and maturation of both bands B and C (Figure 2). However, at higher concentration (100 μM) GSNO inhibited CFTR expression (data not shown). This dose-dependent experiments suggest that concentrations of 5 to 10 μM are optimal for this effect as was shown before[11,13].

### GSNO increases the chloride efflux rate in delF508 CFTR homozygous airway epithelial cell cultures

**Figure 3:**
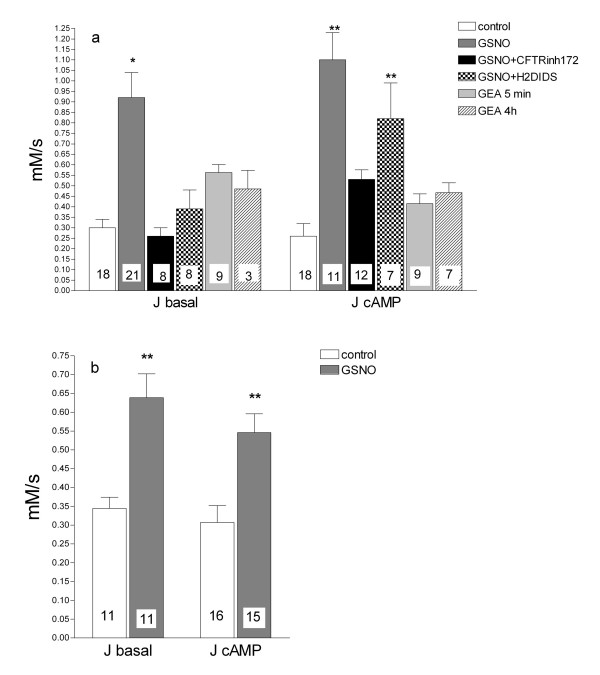
**The effect of GSNO and GEA in CFBE (A) and CFSME (B) cells on the Cl**^-^**efflux rate**. Cells were treated with 60 μM GSNO for 4 hours or 25 μM GEA for the indicated time, then the Cl^- ^efflux rate was measured with the fluorescent indicator MQAE. "***J basal***": the basal efflux rate (recorded during exposure to Cl^-^-free SR without agonists). "***J cAMP***": the cAMP-stimulated efflux rate (recorded during exposure to Cl^-^-free SR containing 20 μM forskolin and 100 μM IBMX). For some experiments, during the basal efflux 400 μM DIDS was used to block Cl^- ^channels (different from CFTR), while the CFTR specific inhibitor CFTR_inh_-172 (40 μM) was used during the cAMP-stimulated efflux. Data are expressed as mean ± SEM and the number of experiments in each group is shown in each column. Significant difference from the control (ANOVA) is indicated by asterisks (* p < 0.05, ** p < 0.01).

Chloride transport studies with the fluorescent dye MQAE revealed that treatment with 60 μM GSNO for 4 hours increased about 3-4-fold the cAMP-dependent efflux rate from 0.26 ± 0.04 to 1.05 ± 0.15 mM/s in CFBE cells (Fig. 3a) and from 0.25 ± 0.06 to 0.6 ± 0.18 mM/s in CFSME cells (Fig. 3b). After GSNO treatment, the basal efflux increased in a similar way as the cAMP-activated Cl^- ^efflux. The basal efflux in CFBE cells was sensitive to 200 μM H_2_DIDS (unspecific blocker of Cl^- ^channels other than CFTR). The cAMP-stimulated efflux in the GSNO-treated cells was effectively blocked by 40 μM CFTR_inh_-172 (a specific blocker of CFTR), which is proof that the CFTR channels were actively involved in this efflux (Fig. 3a).

To further elucidate how much of the increased efflux could be ascribed to NO-release from GSNO, the NO-donor GEA3126 was used. In CFBE cells, treatment with 25 μM GEA3162 for 5 min increased basal Cl^- ^efflux to 0.6 ± 0.04 mM/s which represents about half the effect obtained with GSNO. After 4 hours of GEA3162 treatment Cl^- ^efflux was 0.5 ± 0.09 mM/s, not different from the 5 min treatment. The cAMP-mediated Cl^- ^efflux in these cells was 0.26 ± 0.06 mM/s, which increased to 0.4 ± 0.05 mM/s after 5 min and to 0.5 ± 0.05 mM/s after 4 h of the GEA3162 treatment (Fig. 3a).

**Figure 4:**
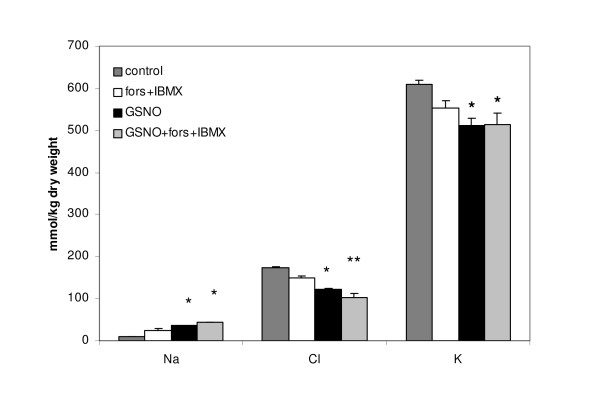
**The effect of GSNO on intracellular ion composition in CFSME cells assessed by X-ray microanalysis method**. "*Control*": Cells kept 2 min in Cl^-^-free SR. "*GSNO*": cells treated with 60 μM GSNO for 4 hours, then exposed for 3 min to Cl^-^-free SR. *fors*+*IBMX *= 3 min exposure to Cl^-^-free SR containing 20 μM forskolin and 100 μM IBMX. Data are expressed as mean ± SEM. Significant difference from the control (ANOVA) is indicated by asterisks (* p < 0.05, ** p < 0.01).

X-ray microanalysis demonstrated that increasing the cellular cAMP concentration with IBMX and forskolin had no significant effect of the cellular chloride or potassium concentration in CFSME cells. Treatment with GSNO (4 h, 60 μM) caused a significant decrease of the cellular chloride concentration, and the decrease for chloride was small but significant in the presence of IBMX and forskolin (Fig. 4).

**Figure 5:**
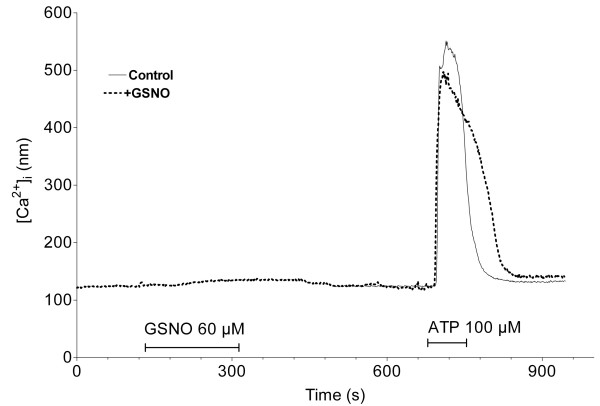
**Plot of the intracellular calcium concentration in the CFBE cells**. In the control experiment (continuous line) the cells were exposed only to ATP (the duration is indicated by the horizontal line). In another experiment (dotted line) the cells were exposed first to GSNO, then, after a 5 minutes washing step, to ATP. The two results were overlapped. Plots represent the averaged results of 3–6 experiments.

In the CFBE cells, 60 μM GSNO had no visible effect on [Ca^2+^]_i_. The effect of 100 μM ATP was a robust elevation in [Ca^2+^]_i _which in the cells previously exposed to GSNO took longer time to return to the baseline values compared to control cells (Fig. 5). Measurement of the area-under-the curve indicates that the total amount of Ca^2+ ^released into the cytoplasm by the ATP exposure was increased by 15% in the case of GSNO pre-treatment (not significant).

## Discussion

Here, we have shown that GSNO increases cAMP-dependent chloride efflux in primary nasal epithelial cells obtained from delF508 homozygous CF patients. As such, it has the potential to represent a corrector therapy for CF. Strikingly, GSNO is an endogenous compound with effects (and therapeutic uses) in several different organ systems. Moreover, GSNO has several different airway effects, and its levels are low in the CF airway; CF and other airways diseases may therefore represent, at least in part, GSNO deficiencies. Additionally, we have 1) confirmed using a monoclonal antibody that GSNO increases maturation of delF508 CFTR in a delF508-homozygous airway epithelial cells line; 2) confirmed that GSNO increases the cAMP-stimulated chloride efflux in different human airway cell lines (CFBE, CFSME) expressing delF508 CFTR by using two independent techniques – the MQAE video-imaging assay and X-ray microanalysis; 3) further characterized the chloride efflux upregulated by GSNO in delF508 CFTR homozygous cells, demonstrating that CFTR is involved; and 4) shown that GSNO does not increase intracellular Ca^2+ ^levels in CFBE cells.

CFTR-dependent Cl^- ^secretion in homozygous CF patients was found to be highly related to the respiratory status in patients with CF with class I and II mutations, and it has been demonstrated that even residual CFTR activity was a good prognostic factor for pulmonary status in this subset of patients [19]. We studied the effect of GSNO on chloride transport in nasal TCC from homozygous delF508 CF patients. The delF508 mutation belongs to class II, which is the class of abnormal protein folding mutations. Chloride efflux mediated by CFTR was, as expected, nonexistent in TCC obtained from CF patients, since these cells lack functional CFTR at the plasma membrane. Treatment of these cells with GSNO for 4 h was able to restore CFTR-mediated chloride transport to a level comparable with the one observed in healthy TCC (Fig. 1).

Due to the scarcity of cells in the nasal brushing biopsy, a direct test of GSNO effect on delF508 CFTR maturation (immunoblot) was not possible in the primary cells. However, in the bronchial cell line CFBE, homozygous for delF508 CFTR, low μM concentrations of GSNO resulted in a dose-dependent increase in the expression of both bands B and C of the immunoblot. These low μM levels of GSNO appear to affect CFTR primarily through a post-transcriptional mechanism, although there appears also to be a modest, transcriptional effect through augmentation Sp3 transcription factor expression and binding [11].

Further, we attempted to characterize the channels involved in Cl^- ^transport using two CF cultured cells models, the CFBE41o- and CFSMEo- cell lines. In CFBE cells, which are homozygous for the delF508 mutation, GSNO treatment resulted in about a tripling of the rate of Cl^- ^efflux both under basal and cAMP-stimulated conditions. A specific blocker of CFTR (CFTR_inh_-172) eliminated more than 50 % of the increased Cl^- ^transport while both GSNO and cAMP agents were present, thus providing evidence of the contribution of CFTR to the observed Cl^- ^efflux. In basal conditions, due to inherent phosphorylation, 60% of wt-CFTR is in activated (open) state. Addition of cAMP increases this state to 100% in wt-CFTR expressing cells[20]. GSNO treatment might result in full activation of CFTR, such that application of cAMP agonists would not increase further the Cl^- ^efflux. It is known that oxidizing conditions slow down CFTR gating while reducing conditions increase open probability [21]. Since GSNO or its products can interact with the cysteine residues of CFTR, modification of gating cannot be excluded. In addition, the CFTRinh-172 is considered a specific blocker and in recent years is widely used to prove the contribution of CFTR. However, a blocker of Cl^- ^channels other than CFTR (H_2_DIDS) also resulted in an inhibition of the efflux, either after GSNO treatment or without such treatment. In the presence of cAMP stimulators a smaller proportion of the Cl^- ^efflux was blocked by non-specific H_2_DIDS compared to the efflux under basal conditions (no cAMP elevating agents present). This implies involvement of CFTR as well as that of other Cl^- ^channels to GSNO-induced increase in Cl^- ^efflux. Besides CFTR, Cl^-^-conductive pathways in bronchial epithelial cells are Ca^2+^-activated and volume-dependent Cl^-^-channels. However, Ca^2+^-activated channels are not likely to be directly responsible for the efflux since the intracellular Ca^2+ ^concentration remained constant after GSNO application (Fig. 5). Volume dependent Cl^- ^channels were not investigated in the current protocol.

The CFSME cells have been shown to produce vestigial amounts of CFTR-mRNA, do not express detectable CFTR protein, and characteristically lack cAMP-induced Cl^- ^currents [22]. We documented Cl^- ^loss after GSNO treatment in CFSME cells by X-ray microanalysis and confirmed this finding by Cl^- ^efflux measurements using the MQAE fluorescent dye method. In comparison with CFBE cells, Cl^- ^efflux in CFSME cells increased to lesser degree after GSNO treatment, which could be due to the fact that these cells are heterozygous for delF508 mutation (delF508/2QX genotype).

Unlike in submucosal airway cell expressing wild-type CFTR (Calu-3) [23] and cerebellar granular cells[24], in CFBE cells there was no elevation in intracellular calcium level after GSNO application. However, we observed an increased area-under-the-curve after ATP exposure subsequent to GSNO treatment. This finding most likely is due to delayed extrusion of Ca^2+ ^from the cytoplasm, which is dependent on the function of either the smooth endoplasmic reticulum Ca-ATPase or the plasma membrane Ca^2+ ^pump. Both are known to be sensitive to oxidation of their thiol groups and free radicals like peroxynitrite, which is one of the possible breakdown products of GSNO.

This paper for the first time demonstrates a GSNO-induced increased Cl^- ^efflux from native CF-airway epithelial cells carrying the delF508 mutation. Both the functional studies and the Western blot experiments support the notion that GSNO in some way helps to overcome the trafficking defect of delF508-CFTR. On the other hand, GSNO does not promote Cl^- ^efflux in airway epithelial cells from healthy persons, which may speak against a direct effect of GSNO on CFTR. In terms of a mechanism by which GSNO induces chloride efflux from cells carrying the delF508 mutation, GSNO has recently been reported to act via both NO-cGMP dependent and independent pathways[25]. We therefore compared Cl^- ^efflux in CFBE cells after GSNO treatment with the effect of a lipophilic NO donor GEA3162, and the slight increase in basal and cAMP-stimulated Cl^- ^efflux caused by GEA3162 may support the notion that the effect of GSNO might to a minor extent be due to NO radical release. However, the mechanism by which GSNO acts is certainly more complex and may well be multifactorial, including effects at the nuclear level [11]. Nevertheless, the fact that GSNO is able to normalize Cl^- ^efflux from CF airway epithelial cells, in combination with the fact that GSNO is a naturally occurring, non-toxic compound, supports further study of this compound as a potential drug for CF.

## Conclusion

We have shown that treatment with GSNO normalizes Cl^- ^efflux from cells isolated from CF patients with the delF508-mutation; and 2) associated increase in Cl^- ^efflux found was in part due to CFTR maturation from respiratory epithelial cell lines with the delF508-CFTR mutation. This endogenous compound hence may be of value as a possible candidate to correct the basic defect in CF.

## Abbreviations

GSNO- S-nitrosoglutathione

CFTR- cystic fibrosis transmembrane conductance regulator

CF- Cystic Fibrosis

CFBE- CF human bronchial epithelial cell line CFBE41o- homozygous for delF508 cystic fibrosis mutation

CFSME- CF submucosal epithelial cell line CFSMEo- with the genotype delF508/4QX

CFTR_inh_-172- specific thiazolidinone CFTR inhibitor

H_2_DIDS- 4,4'-diisothiocyanatodihydrostilbene-2,2'-disulfonic acid

TCC- tall columnar ciliated

MQAE- N-(ethoxycarbonylmethyl)-6-methoxyquinolinium bromide

GEA3162- 5-amino-3-(3,4-dichlorophenyl)1,2,3,4-oxatriazolium

IBMX- 3-isobutyl-1-methylxantine

## Competing interests

The author(s) declare that they have no competing interests.

## Authors' contributions

ZS took the nasal biopsies, carried out studies with the fluorescent Cl^- ^indicator, X-ray microanalysis, performed statistical analysis and drafted the manuscript; AD took the nasal biopsies, carried out studies with the fluorescent Cl^- ^indicator, performed statistical analysis; JK carried out the studies with the fluorescent Ca^2+ ^indicator; KZ performed immunoblot analysis; LH was responsible for the CF-patients; BG, GMR and AD planned and supervised the study. All authors took part in the writing of the manuscript.
